# Interplay of PKD3 with SREBP1 Promotes Cell Growth via Upregulating Lipogenesis in Prostate Cancer Cells

**DOI:** 10.7150/jca.31254

**Published:** 2019-10-19

**Authors:** Ling Li, Liang Hua, Huihui Fan, Yu He, Wanfu Xu, Lin Zhang, Jie Yang, Fan Deng, Fangyin Zeng

**Affiliations:** 1 Department of Clinical Laboratory, The Fifth Affiliated Hospital, Southern Medical University, Guangzhou, China; 2 Center Laboratory, Guangzhou Women and Children's Medical Center, Guangzhou Medical University, Guangzhou, China; 3 Department of Cell Biology, School of Basic Medical Sciences, Southern Medical University, Guangzhou, China; 4 Department of Clinical Laboratory, Foshan women and children hospital, Foshan, China

**Keywords:** fatty acid synthesis, lipid metabolism, protein kinase D3, prostate cancer, SREBP1

## Abstract

Protein kinase D (PKD) has been implicated in cancer cell survival, proliferation, migration and angiogenesis. However, it is still unknown whether PKD regulates cell proliferation through lipid metabolism in cancer cells. Here we report a novel function of PKD3, a member of PKD family, in regulating of prostate cancer cell proliferation by modulation of SREBP1-mediated *de novo* lipogenesis. We show that silencing of PKD3 significantly reduces lipid content and expression of the lipogenic genes encoding FASN and ATP-citrate lyase (ACLY). Moreover, endogenous PKD3 interacts with sterol regulatory element binding protein 1(SREBP1) in DU145 cells. Interestingly, PKD3 silencing decreases not only the level of matured-SREBP1 (68KD) but also the binding of SREBP1 to the promoter of *fasn* gene. In addition, overexpression of SREBP1 reverses the suppression of cell growth caused by PKD3 depletion. Finally, immune-histochemical staining indicate that PKD3 expression is positively correlated with expression of FASN and SREBP1 in prostate cancers. Taken together, these data suggest that targeting PKD3-mediated *de novo* lipogenesis may be a potential therapeutic approach to block prostate cancer progression.

## Introduction

The reprogramming of cellular energy metabolism is one of the recognized ''emerging hallmarks'' of human cancer 1. Enhanced aerobic glycolysis (Warburg effect) is the main metabolic characteristics of malignant tumors 1, 2. Accumulating evidence showed that metabolic rewiring in cancer not only limited to glucose metabolism, but also including lipid and amino acids 3, 4.

Lipid metabolism is altered in rapidly proliferating cells, which synthesize lipids through *de novo* lipogenesis 5-7. Continuous *de novo* lipogenesis provides cancer cells with membrane building blocks, signaling lipid molecules and post-translational modifications of proteins to support rapid cell proliferation 8, 9. The expression and activity of key enzymes involved in *de novo* fatty acid synthesis, such as ATP citrate lyase (ACLY), acetyl-CoA carboxylase (ACC) and fatty acid synthase (FASN), are upregulated and associated with poor clinical outcomes in various types of cancer^7^, ^10^, ^11^. Moreover, overexpression of sterol regulatory element-binding proteins (SREBP1s), a key transcription factor that regulates transcription of key enzymes in *de novo* lipogenesis, was also observed in human cancer tissues and correlated with progression of various cancers 12-14. However, mechanisms underlying the increased lipogenesis in cancers are not completely understood.

PKD belongs to a family of serine/threonine protein kinases that comprises of three members, namely PKD1 (PKCμ), PKD2 and PKD3 (PKCν). PKD has been implicated in many biological processes including cell proliferation 15, cell migration 16, angiogenesis 17, epithelial to mesenchymal transition (EMT) 18 and stress-induced survival responses 19. Altered PKD expression and activity have been implicated in aspects of tumorigenesis and progression, including survival, growth and invasion 15, 20, 21. We have previously demonstrated that PKD plays an important role in the survival and tumor invasion of prostate cancer and targeted PKD inhibition potently blocks cell proliferation and invasion in prostate cancer cells 22, 23. Currently, we have also showed that PKD contributed to tumor angiogenesis through mast cells recruitment and upregulation of angiogenic factors in prostate cancer microenvironment 24. However, whether PKDs regulate de novo lipogenesis in the tumor cells remains unknown.

In this study, we explored the role of PKD3 in the de novo lipogenesis of prostate cancer cells. We demonstrated that PKD3 contributes to the lipogenesis through regulating SREBP1-mediated* de novo* lipogenesis and proliferation of prostate cancer cells.

## Materials and Methods

### Cell culture, siRNA and plasmid transfections

The human prostate cancer cell lines DU145 and PC3 were obtained from ATCC. All the cell lines were cultured in DMEM medium (Gibico) supplemented with 10% fetal bovin serum and 100 units/mL penicillin/streptomycin in an atmosphere of 5% CO_2_ at 37° C. Cells were plated into 6-well plates and transfected with 120nM siRNA duplexes (GenePharma, Suzhou) using Lipofectamine 3000 (Invitrogen) according to the manufacturer's protocol. The siRNA duplexes were as follows: siPKD3: 5'-GAACGAGUCUUUGUAGUAATT-3' (Silencer Selected Validated siRNA, catalog no.4390824), siFASN: 5'-GAGCGUAUCUGUGAGAAACtt-3', siFASN generated as described 25. Flag, flagSREBP1c plasmid (Addgene, Cambridge, USA) were transfected using Hilymax from Dojindo (Kamimashikigun, Kumamoto, Japan) according to the manufacturer's protocol.

### RNA extraction and real-time quantitative PCR analysis (RT-qPCR)

RNA was extracted from prostate cancer cells using Trizol reagent (Takara, Dalian, China). Reverse transcription were carried out using the PrimeScript RT reagent kit(Takara) and mRNA level was determined by SYBR Green PCR Master Mix (Takara) according to the manufacturer's protocol. The RT-qPCR primers were as follows: PKD3 forward, 5'-CTGCTTCTCCGTGTTCAAGTC-3' and reverse, 5'-GAGGCCAATTTGCAGTAGAAATG-3'; SREBP1 forward, ACAGTGACTTCCCTGGCCTAT and reverse, 5'-GCATGGACGGGTACATCTTCAA-3'; FASN forward, 5'-AAGGACCTGTCTAGGTTTGATGC-3' and reverse, 5'-TGGCTTCATAGGTGACTTCCA-3'; ACLY forward, 5'-TCGGCCAAGGCAATTTCAGAG-3' and reverse 5'-CGAGCATACTTGAACCGATTCT-3'; β-actin forward, TGGCACCCAGCACAATGAA and reverse, 5'-CTAAGTCATAGTCCGCCTAGAAGCA-3'.

### Co-immunoprecipitation (Co-IP) and Immunoblotting

Co-immunoprecipitation and immunoblotting were performed as described in our previous studies 22. For western blot analysis, prostate cancer cells were plating in six wells plate. After 48-hours transfection with the indicated siRNAs, the cells were lysed by loading buffer containing proteinase inhibitors and phosphatase inhibitors. Cytoplasmic and nuclear extracts were obtained with Nuclear and Cytoplasmic Protein Extraction kit (Beyotime Institute of Biotechnology, China) according to the manufacturer's instructions. The protein concentration was determined using Bradford reagent (Keygen Biotech, Jiangsu, China) or enhanced BCA protein assay kit (Beyotime Institute of Biotechnology, China). The cell lysates were electrophoresed on 10% SDS-PAGE and transferred onto polyvinylidene difluoride membranes (Millipore, Charlottesville, VA, USA), then incubated overnight at 4℃ with primary antibodies against PKD3(#5655, Cell Signaling Technology), SREBP-1(sc-13551, SantaCruz), SREBP1(sc-366, SantaCruz), polyclonal FASN(A6273, Abclonal), ACLY(#13390, Cell Signaling Technology), GAPDH(RM2007, Beijing Ray), TBP(A2192, Abclonal), respectively. The blots were incubated with goat anti-rabbit or anti-mouse secondary antibodies (Ray, Beijing, China), visualized using a chemiluminescence method (Western Lightning Plus kit, Perkin Elmer).

### Immunofluorescence

PC3 or DU145 cells were transiently transfected with control or PKD3 siRNAs for 36 hours, cells were washed with PBS three times, fixed with 4% buffered formalin for 20 minutes at room temperature, permeabilized with 0.5% Triton X-100 for 30 minutes, and then blocked for 1 hour in CAS block buffer. Cells were incubated with mSREBP1 (1:200) primary antibodies overnight in wet box. Cells were then washed with PBS three times and incubated with rabbit TRITC secondary antibody for 1 hour. After washed with PBS and nuclei stained with DAPI, cell images were captured at 600× magnification under a laser scanning confocal fluorescent microscope.

### Oil Red O Staining

Cells were transfected with indicated siRNA for 24 hours, seeded on coverslips in 24-well plates and washed twice with PBS after 48 hours. Then, the cells were fixed with 4% formalin (Tianjun, Guangzhou) at room temperature for 1 hour, washed with distilled water twice and 60% isopropanol at room temperature for 5 min after removing formalin. Cells were dried at room temperature and stained with 3% Oil Red O working solution (Oil red O in 100% isopropanol: distilled water=3:2) for 20 min at 37℃. Images were captured at 400× magnification under a microscope.

### Adipored assay

Adipored assay were measured the intracellular lipid content using the reagent kit (Lonza). In brief, after transfected with siRNA 24 hours, 50000 cells were digested and seeded to 24-well plates, and assayed for the quantification of intracellular lipid content according to the Adipored assay reagent kit manufacture's protocol after 48 hours. The experiments were performed in sixth and standardized with cell number measured by CCK8 kit.

### Immunohistochemistry (IHC) of human tissue microarrays

A human prostate cancer tissue microarray containing 80 histologically confirmed prostate cancer samples, 8 adjacent normal prostate tissues and 8 prostate tissues was purchased from Alenabio (Cat.No.PR1921a; Alenabio Biotechnology, China). Detailed clinical characteristics and criteria of prostate cancer patients from tissue microarray samples were provided by Alenabio (http://www.alenabio.com/public/details?productId=29885&searchText=PR1921a) and shown in Supplementary Table S1. Prostate cancer samples were immune-stained against the primary antibodies of rabbit anti-mSREBP1(sc-366, SantaCruz), rabbit anti-polyclonal FASN(A6273, Abclonal), mouse anti-PRKD3(WH0023683M1, Sigma), respectively , as described in our previous studies^24^. Briefly, the slides were dewaxed and rehydrated in distilled water, sections were immersed in citrate buffer (C_6_H_5_Na_3_O_7_·2H_2_O) and then microwaved for 20 min for antigen retrieval. Endogenous peroxidase activity was blocked with 0.5% (v/v) H_2_O_2_. The slides were transferred into a humidified chamber, incubated with 5% (v/v) horse serum for 30 min and then incubated with indicated primary antibodies overnight at 4° C. After primary antibody incubation, the slides were immersed in peroxidase-labeled secondary antibody for 30 min at room temperature. Immunostaining scores were estimated according to the staining intensity and the number of imunopositive cells by two experienced pathologists as described previously 26. Immunohistochemical scores of 4-12 and scores of ≤3 were defined as low and high expression, respectively.

### Luciferase reporter assay

Cells were transiently co-transfected with the PKD3 siRNA, FAS promoter luciferase plasmid and internal control plasmid (pGL4.74[hRluc/TK],from Promega) to measure the FAS promoter activity using the luciferase assay kit from Promega. FAS promoter luciferase was a gift from Bruce Spiegelman (Addgene plasmid # 8890; http://n2t.net/addgene:8890; RRID: Addgene_8890).Thirty hours after transfection, firely and Renilla luciferase activities were determined as described by the manufacturer instruction (Promega). These methods were performed as described in our previous studies 22.

### Cell proliferation assay

For cell proliferation assay, we used Cell Counting Kit-8(CCK8) kit (Dojindo Laboratories, Kumamoto, Japan) according to the manufacturer's protocol. 24 hours after transfected with specific siRNA, we digested cell lines, seeded to 96-wells plates and measured the absorbance at each time point. All of the experiments were performed in fifth.

### Statistical analysis

All data were analyzed with GraphPad Prism5.0 software and SPSS 17.0 software. Two-tailed Student's t-test were used to calculate. *p<0.05, **p<0.01, and ***p<0.001 were considered statistically significant. Spearman rank test was used to evaluate the correlation between the PKD3 expression and FASN expression or SREBP1 expression.

## Results

### PKD3 silencing inhibits lipid content in prostate cancer cells

**Figure 1:**
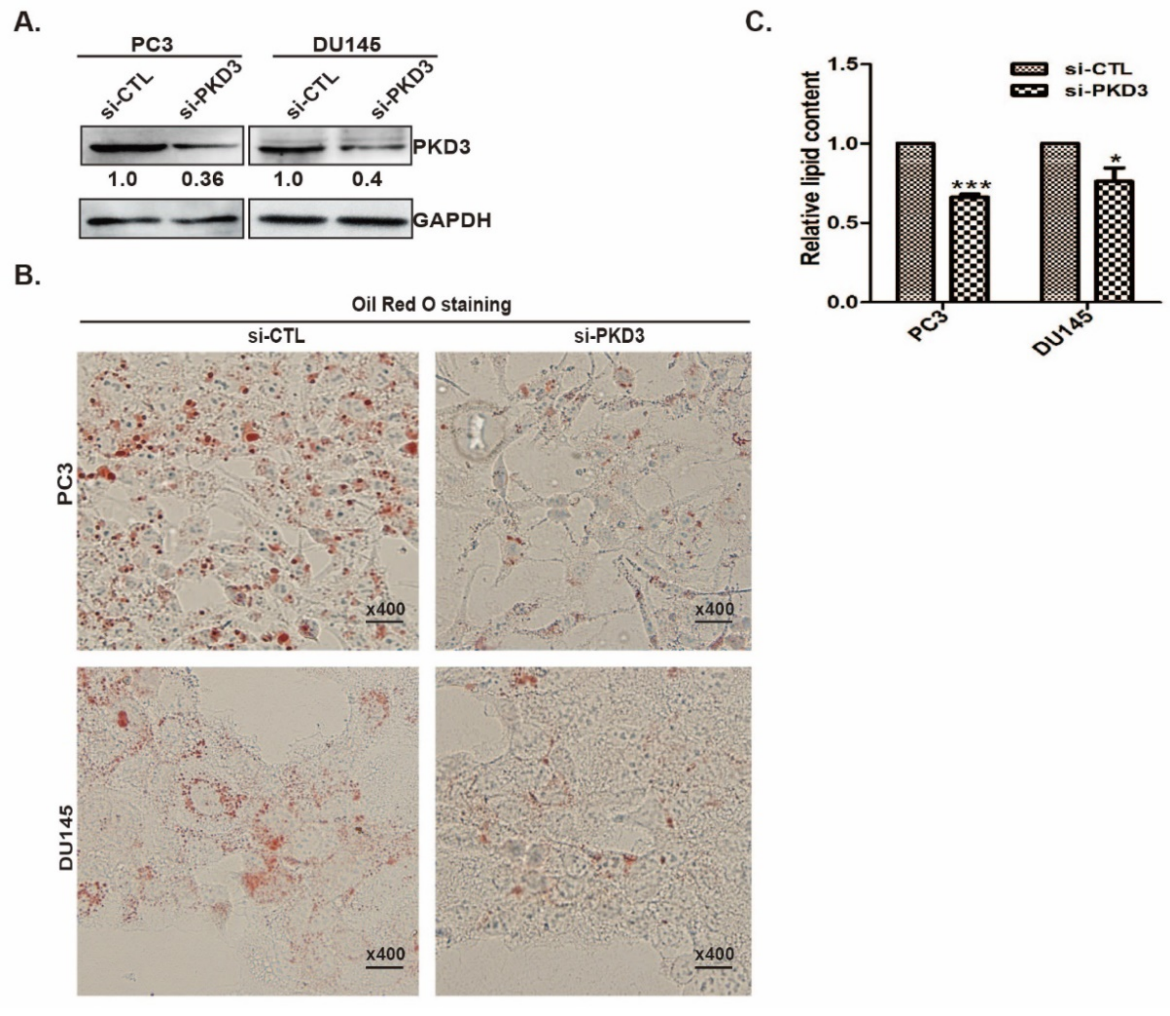
**Knockdown of PKD3 decreased lipid droplet contents in PC3 and DU145 cells. A**. PC3 or DU145 cells were transiently transfected with control or PKD3 siRNA (si-PKD3) for 60 hours. Cells were lysed and analyzed by Western blotting with anti-PKD3 antibodies or anti-GAPDH antibody; **B.** PC3 or DU145 cells were transfected as A, then stained with Oil red O and depicted at 400x magnification; C. Adipored assay were performed to quantify intracellular lipid content within cells transiently transfected with control or PKD3 siRNAs in 24-well plate after 60 hours. Lipid content was normalized with respective cell number for each group. Data represent the mean ± S.D. of three independent experiments. *p<0.05, **p<0.01 and ***p<0.001.

Our previous study showed that PKD3 promotes prostate cancer cell survival and invasion 22, 27. To explore whether PKD3 was involved in lipid metabolism, we assessed the neutral lipid droplet content of prostate cancer cells by Oil red O (ORO) staining that mainly stains cellular cholesterol esters and neutral triglycerides. We first transiently transfected prostate cancer PC3 or DU145 cells with siRNA control (si-CTL) or siRNA of PKD3. Silencing of PKD3 were verified by Western blotting (Fig 1A). Oil red O staining shown that knockdown of endogenous PKD3 decreased cellular cholesterol esters and neutral triglycerides in PC3 or DU145 cells (Fig 1B). We then used Adipored reagent to stain intracellular lipid droplets. As shown in Fig 1C, cellular lipid droplets were also significantly reduced in PC3 and DU145 cells transfected with siRNA of PKD3 compared with cells transfected with siRNA of control. These results suggested that PKD3 may play role in de novo lipogenesis of prostate cancer cells.

### Lipogenesis enzymes and transcriptional activity of FASN are inhibited by PKD3 depletion in prostate cancer cells

**Figure 2:**
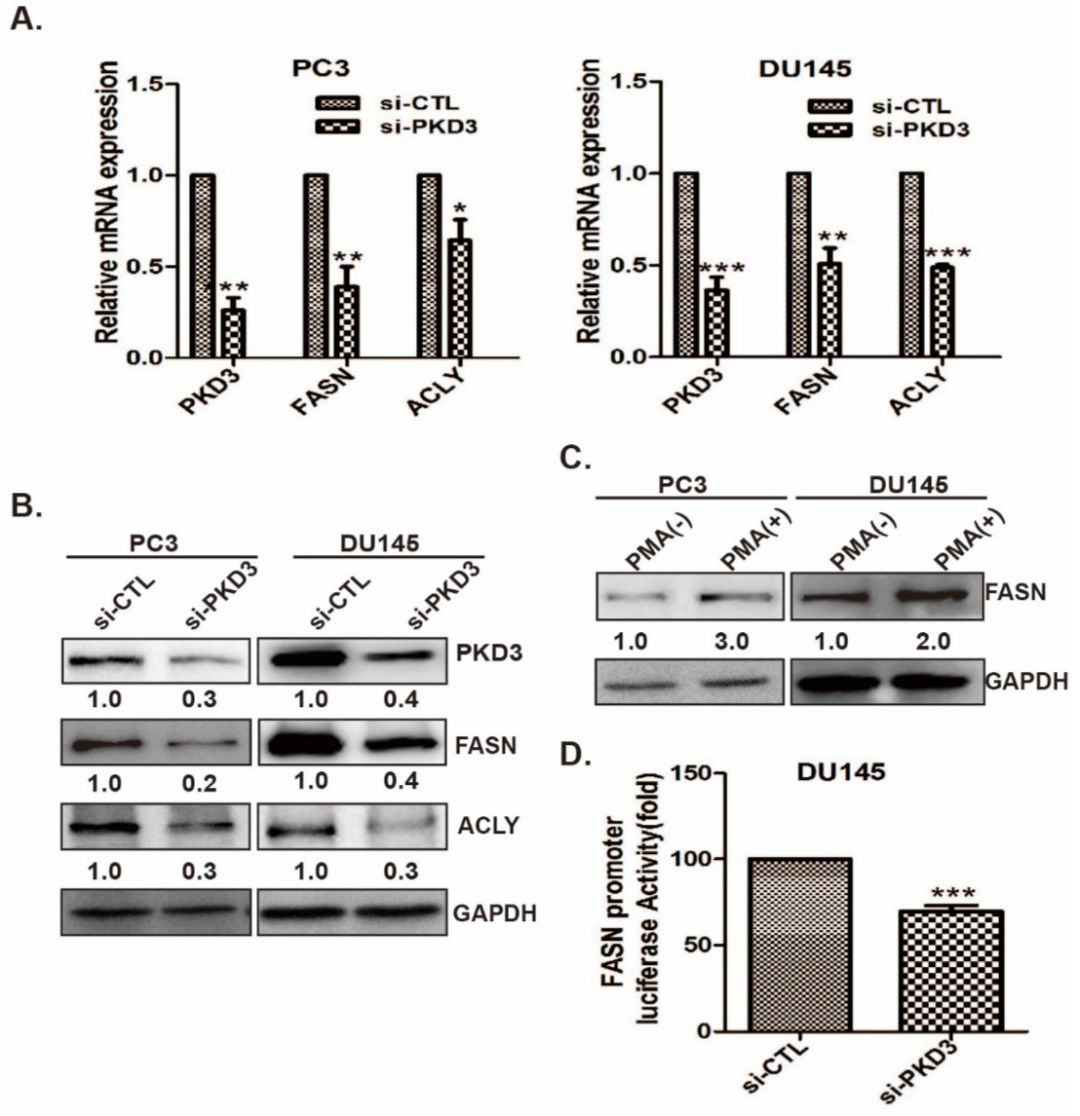
**Silencing of PKD3 reduced key enzyme expression of** *de novo*** lipogenesis and *fasn* transcriptional activity. A**.PC3 or DU145 cells were transiently transfected with control or PKD3 siRNAs. After 48 hours, mRNA levels of critical lipogenesis enzymes FASN and ACLY were analyzed by RT-qPCR. Data were shown as mean ± S.D. of three independent experiments. *p<0.05, **p<0.01 and ***p<0.001; **B**. PC3 or DU145 cells were transfected as indicated, cells were lysed 60 hours post transfection for immunoblotting of FASN/ACLY proteins as well as GAPDH (internal control); **C.** PC3 and DU145 cells were treated with 100nM PMA for 48h after serum starved for 24h, cells were lysed and immunobloted with anti-FASN antibodies; **D.**DU145 cells were co-transfected with the PKD3 siRNA, FAS promoter luciferase plasmid and internal control plasmid. After 48h, FASN luciferase activity were analyzed. Data were shown as mean± S.D. of three independent experiments. *p<0.05, **p<0.01, and ***p<0.001.

Since knockdown of PKD3 decreases the lipid droplet content in prostate cancer cells, we examined the effect of PKD3 on the expression of key enzymes in the *de novo* lipogenesis of prostate cancer cells. As shown in Fig 2A, depletion of PKD3 led to significant down-regulation of mRNA level of FASN and ACLY, two key enzymes in *de novo* lipogenesis, compared to non-targeting siRNA control in PC3 or DU145 cells. Similarly, protein levels of FASN and ACLY were also reduced in PC3 or DU145 cells transfected with siRNA of PKD3 compared with that siRNA control cells (Fig 2B). As phorbol 12-myristrate 13-acetate (PMA) was shown to be able to activate PKC/PKD family 15, 27, we explored whether PMA induced FASN expression in prostate cancer cells. Western blotting showed that FASN expression was enhanced in response to PMA treatment in PC3 and DU145 cells (Fig 2C).

To further confirm the possibility that PKD3 contributes to FASN expression via upregulating *fasn* gene promoter transactivation, we then co-transfected *FAS* reporter luciferase plasmid with *Renilla* reporter luciferase plasmid (internal control reporter) in DU145 cells transiently transfected with siRNA control or siRNA PKD3. Dual luciferase reporter assay showed that depleting of PKD3 significantly reduced *fasn* transactivation in DU145 cells compared with siRNA control group (Fig 2D), indicating that PKD3 are involved in FASN expression through regulating transcriptional activity of *fasn* gene.

### Endogenous PKD3 interacts with SREBP1 and increases the mSREBP1 level and nuclear entry

**Figure 3:**
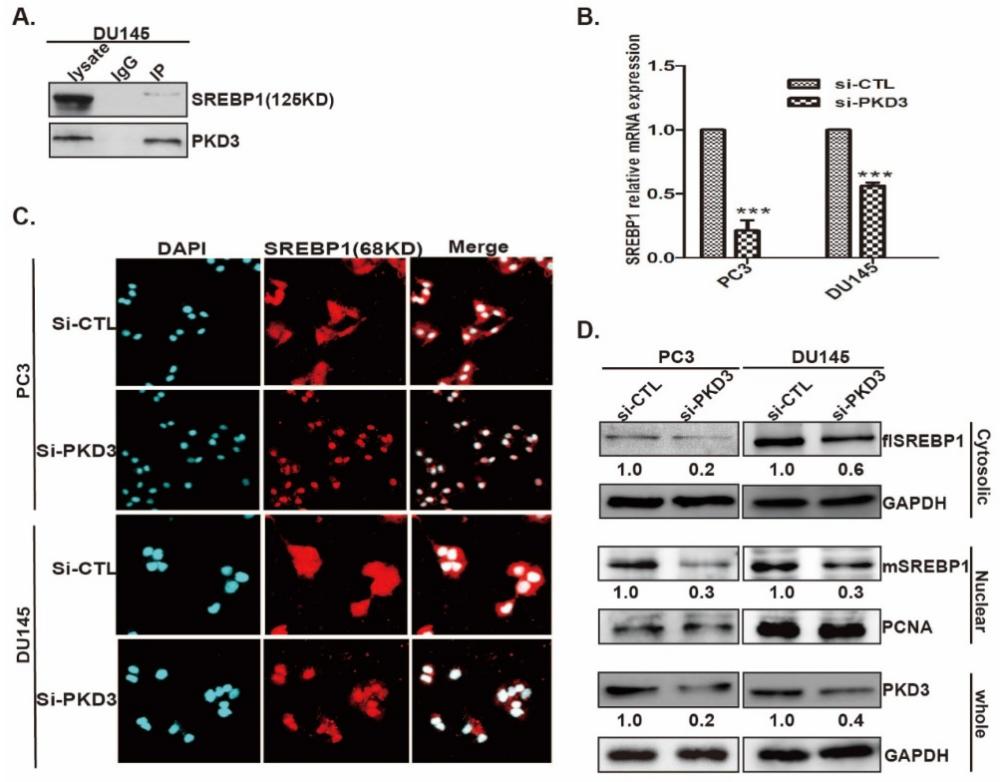
** PKD3 interacts with SREBP1 and regulates expression and nuclear entry of mSREBP1 (68KD) in prostate cancer cells. A.** DU145 cells were grown to 70-80% confluence, whole lysates were immunoprecipitated with PKD3 antibody and co-precipitates with SREBP1 were detected by immunoblotting; **B.**PC3 and DU145 cells were transiently transfected with control or PKD3 siRNAs. After 48 hours, mRNA levels of SREBP1 were analyzed by RT-qPCR. Data are shown as mean ± S.D. of three independent experiments. *p<0.05, **p<0.01, and ***p<0.001; **C.**PC3 and DU145 cells were transiently transfected with control or PKD3 siRNAs. After 36 hours, confocal microscopy images (at 600×) showing SREBP1 (Red-TRITC) in PC3 and DU145 cells. DAPI (in blue) stains nuclei; **D.** PC3 and DU145 cells were transfected as indicated, cells were lysed 60 hours post transfection for immunoblotting of the cytosolic and nuclear SREBP1, the filters were then blotted with anti-GAPDH or anti-PCNA as loading controls.

Current data showed that SREBP1 is the key regulator of lipogenesis related genes expression 28. We further clarified the interaction of PKD3 with SREBP1 and the effect of PKD3 on SREBP1 expression and nuclear entry. As shown in Fig 3A, immunoprecipitation assay demonstrated that endogenous PKD3 interacted with SREBP1 in DU145 cell. Given that the precursor form of SREBP1(125KD) is bound to the ER membrane and the mature form of SREBP1(68KD) translocates to the nucleus during starvation or sterol depleted condition 29, we investigated whether PKD3 regulates the formation of mature SREBP1(mSREBP1) and nuclear entry in prostate cancer cells. Real time RT-PCR indicated that silencing of PKD3 remarkably decreased the mRNA level of SREBP1 in PC3 and DU145 cells compared with that in the siRNA control group (Fig 3B). Moreover, immunofluorescence staining showed that nuclear entry of mSREBP1(68KD) were significantly decreased with PKD3 depletion in PC3 and DU145 cells compared with siRNA control in both of cells (Fig 3C). In addition, cells fractionation and Western blotting also demonstrated that silencing of PKD3 decreased the cytosolic (full length SREBP1, flSREBP1) and nuclear SREBP1(mSREBP1) in PC3 and DU145 cells (Fig 3D). Collectively, these data indicated that PKD3 contributes to *de novo* lipogenesis via interaction with SREPB1, promoting the nuclear entry and expression of SREBP1 in prostate cancer cells.

### PKD3 promotes cell growth through SREBP1 mediated *de novo* lipogenesis

**Figure 4:**
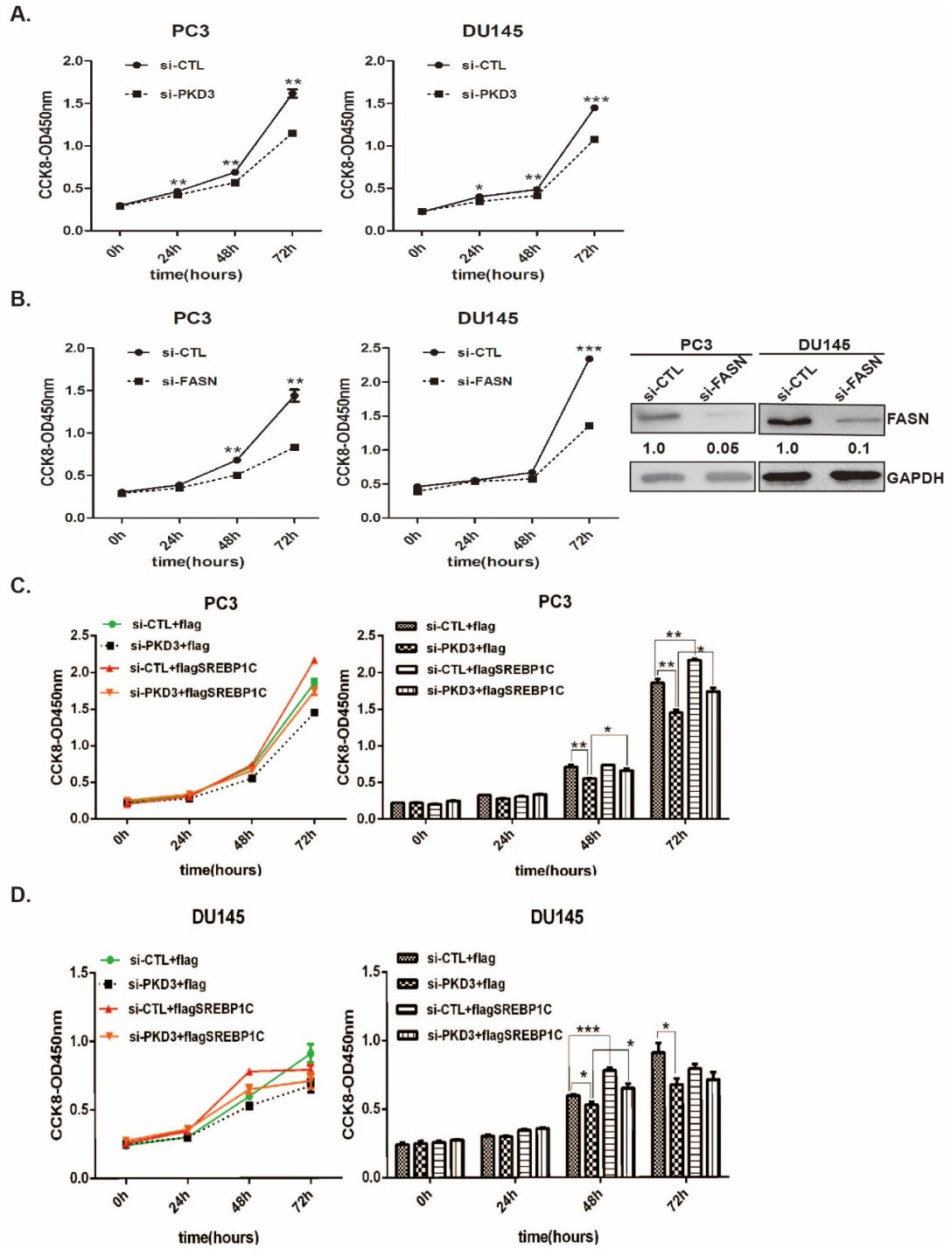
** Overexpression of SREBP1c reverses cell growth inhibition triggered by PKD3 silencing in prostate cancer cells. A.** PC3 and DU145 cells were transiently transfected with control or PKD3 siRNAs. After 24 hours transfection, the cell viability were measured by CCK8 kit at point of 0, 24, 48 and 72 hours; **B.** PC3 and DU145 cells were transiently transfected with control or FASN siRNAs, the cell viability were measured as described in **A**. Immunoblot analysis were measured to verify sufficient knockdown; **C-D.** SREBP1C plasmids were transfected in PC3 or DU145 cells after depletion of PKD3 for 24 hours, the cell viability were measured as **A-B**. Data are shown as mean ± S.D. of three independent experiments. *p<0.05, **p<0.01, and ***p<0.001.

To further validate the contributions of PKD3 to prostate cancer cell growth through SREBP1-mediated lipogenesis, we first investigated the effect of PKD3 and FASN on the prostate cancer cell growth by cell proliferation assay. As shown in (Fig 4A-B), knockdown of PKD3 or FASN dramatically inhibited cell growth in PC3 and DU145 cells compared with siRNA control. Moreover, we contransfected siRNA of PKD3 with SREBP1c plasmid into PC3 or DU145 cells. Compared with control plasmid (flag-pcDNA3, flag), overexpression of SREBP1c remarkably reversed cell growth inhibited by PKD3 silencing in PC3 or DU145 cells (Fig 4C-D). These results indicated that PKD3 promoted tumor cell growth through SREBP1 mediated *de novo* lipogenesis in prostate cancer cells.

### PKD3 is positively correlated with expression of FASN and mSREBP1 in prostate cancer tissues

**Figure 5:**
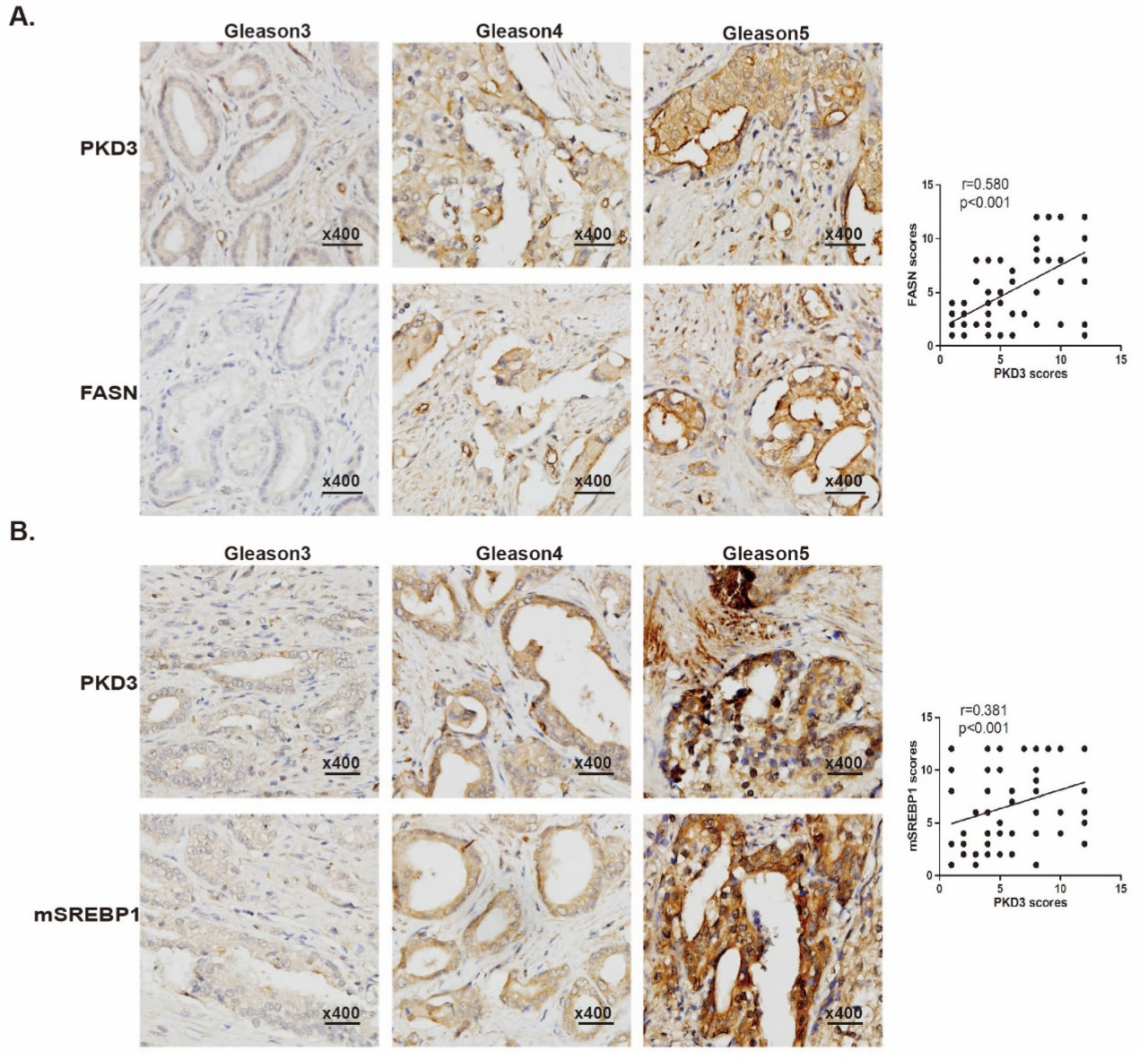
** PKD3 expression is positively correlated with expression of FASN and mSREBP1 in prostate cancer tissues.** Tissue microarray slides containing 80 prostate cancer samples, 8 adjacent normal prostate tissue and 8 prostate tissue were subjected to IHC analysis for PKD3, FASN and mSREBP1. **A**. Representative images depicted at 400x magnification for PKD3 and FASN antibody staining of different Gleason scores are shown (left panels). Spearman rank test were used to study the correlations between PKD3 and FASN expression. (r=0.580, p<0.001) (right panels). **B**. Representative images depicted at 400x magnification for PKD3 and mSREBP1 antibody staining of different Gleason scores are shown (left panels), Spearman rank test were used to study the correlations between PKD3 and mSREBP1 expression. (r=0.381, p<0.001) (right panels).

We have previously demonstrated that expression of PKD3 was elevated in human prostate cancer tissues 27, other data also showed that expression of FASN was correlated with the progression of prostate cancer as well as pathologic stage 30, 31. To evaluate the correlation of PKD3 with expression of FASN and matured-SREBP1 (mSREBP1) as well as pathological progression of prostate cancer, we examined the expression of PKD3, FASN and mSREBP1 by immune-histochemical staining in a total of 80 prostate cancer specimens and 16 normal tissue cores, among which were 8 normal tissues adjacent to the tumors. Detailed clinical and pathological characteristics of prostate cancer patients from tissue microarray samples were listed in Supplementary Table S1. As shown in Fig. 5A, expression of PKD3 was positively correlated with FASN expression (r=0.580, *p*<0.001) and Gleason score. These results recapitulated the functional link between PKD3 and FASN in the progression of prostate cancer. Furthermore, we also examined the correlation of PKD3 expression and mSREBP1 levels in prostate cancer. Results showed that PKD3 expression also positively correlated with mSREBP1 expression as well as Gleason score (r=0.381, *p*<0.001) (Fig 5B).

## Discussion

An enhanced capacity of lipid synthesis in cancer cells has long been recognized as an important characteristic of malignant tumors 5, 32. Although expression of PKD3 has been shown to promote prostate cancer cell growth and invasion 22, 23, the role of PKD3 was never been uncovered in lipid metabolism alteration of cancer cells so far. In this study, we demonstrated that PKD3 contributes to cell proliferation by modulation of SREBP1-mediated expression of FASN and ACLY as well as* de novo* lipogenesis in prostate cancer cells. Furthermore, we showed overexpression of SREBP1 reverses the cells growth inhibition induced by PKD3 depletion in prostate cancer cells. Finally, clinical data also showed that PKD3 expression was positively correlated with FASN and SREBP1 expression as well as Gleason scores in prostate cancer. These results indicated that PKD3 promoted the tumor cell growth via SREBP1-mediated *de novo* lipogenesis in prostate cancer cells.

Increased* de novo* lipogenesis has been implicated in numerous types of cancer and has been demonstrated to one of the main characteristics of cancer regardless of the extracellular lipid availability 7. Elevated level of SREBP1, one of the key transcription factors that regulate the transcription of lipogenic enzymes such as FASN and ACLY, has been found in numerous types of cancer 7. Previous studies have been showed that maturation and nuclear translocation of SREBP-1c are regulated by a variety of proteins. For example, oncogenic PI3K and KRAS mediated-mTORC1 activation promote nuclear accumulation of mature SREBP1 in breast cancer cell 33. Meanwhile, the other data showed that SREBP1 was also activated by Akt mediated -mTORC1 signaling 34-35. Moreover, HBV protein X interacts with LXRa (liver X receptor alpha) and enhances the binding of LXRa to LXRE (LXR-response element), thereby resulting in the up-regulation of SREBP1 and transactivation 36. In addition, AMPK suppressed SREBP-1c proteolytic cleavage and nuclear translocation by Ser372 phosphorylation 37, 38. Conversely, AMPK inhibition resulted in activation of SREBP1 and upregulated lipid synthesis 39. Although the apparent importance of SREBP1-mediated* de novo* lipogenesis in cancers, it is unclear whether other proteins contribute to the tumor cell growth via *de novo* lipogenesis.

One of the interesting findings is that PKD3 promotes lipid synthesis by regulation of SREBP1 expression and nuclear entry as well as FASN and ACLY expression in prostate cancer cells. We found endogenous of PKD3 interacts with SREBP1 in prostate cancer cells. PKD3 silencing not only decreased the formation of mature SREBP1(mSREBP1) and nuclear entry, but also inhibited expression of SREBP1, FASN and ACLY. Moreover, luciferase assay also demonstrated that deletion of PKD3 significantly reduced the binding of SREBP1 to the promoter of *fasn* gene in DU145 prostate cancer cells. Given that PKD3 promotes *de novo* lipogenesis and cell growth in prostate cancer cells, we further explored whether overexpression of SREBP1 will rescue the cell growth inhibition caused by knockdown of PKD3. Cell proliferation assay demonstrated that overexpression of SREBP1 remarkably reversed the inhibitory effect of PKD3 silencing on cell growth in DU145 and PC-3 cells, providing a key link between PKD3-mediated *de novo* lipogenesis through modulation of SREBP1 and cell proliferation in prostate cancer cells.

Overexpression of SREBP1 was observed in human prostate cancer tissues, which are related with progression to androgen-refractory/castration-resistant disease 13, 14. In the present study, we also showed that higher expression of PKD3, FASN, mSREBP1 were observed in malignant tumors of the prostate and correlated with Gleason scores, which strengthen the results that PKD3 regulating lipid synthesis via targeting SREBP1 in prostate cancer cells. However, the functional consequence of this intracellular connection should be fully understood in animals and clinical cohort study.

In conclusion, our findings indicated that a novel function of PKD3 that promotes cell proliferation by modulating SREBP1 and *de novo* lipogenesis in prostate cancer cells. The interplay of the PKD3 with SREBP1 and lipogenesis pathways in regulating lipid metabolism may have important implications in prostate cancer development and potentially provide novel molecular targets for prevention and treatment of the cancer.

## Supplementary Material

Supplementary figures and tables.

## Abbreviations

PKD: protein kinase D
SREBP1: sterol regulatory element binding protein 1
FASN: fatty acid synthase
ACLY: ATP citrate lyase
ACC: acetyl-CoA carboxylase
