# Supplementary material for: Interplay of PKD3 with SREBP1 Promotes Cell Growth via Upregulating Lipogenesis in Prostate Cancer Cells
Source: J Cancer. 2019 Oct 19;10(25):6395–404. doi: 10.7150/jca.31254 (PMC6856730; doi:10.7150/jca.31254)
Supplement: Supplementary file 1 — Supplementary figures and tables. [file jcav10p6395s1.pdf]

Supplementary Table S1：Clinical characteristics of prostate cancer from tissue microarray samples

No.	Age ranges	Pathology diagnosis	TNM	Stage	Type	Gleason Grade	PSA
1	70-80	Adenocarcinoma(sparse)	T2AN0M0	II	Malignant	4	+
2	70-80	Adenocarcinoma	T2N0M0	II	Malignant	3	++
3	60-70	Adenocarcinoma	T3N1M1	IV	Malignant	3	+
4	70-80	Adenocarcinoma	T2N0M0	II	Malignant	4	+
5	70-80	Adenocarcinoma	T2N1M0	-	Malignant	4	+
6	60-70	Adenocarcinoma	T2N0M0	II	Malignant	4	++
7	60-70	Adenocarcinoma	T2N0M1	IV	Malignant	4	+
8	60-70	Adenocarcinoma	T2N0M0	-	Malignant	-/3	++
9	70-80	Adenocarcinoma	T2N1M1C	IV	Malignant	4	+
10	70-80	Adenocarcinoma	T2N0M0	IIA	Malignant	4	++
11	60-70	Adenocarcinoma	T2N0M0	II	Malignant	4	+
12	70-80	Adenocarcinoma	T2N0M0	II	Malignant	4	++
13	70-80	Adenocarcinoma	-	-	Malignant	4	+
14	50-60	Adenocarcinoma	T2N0M0	II	Malignant	3	+
15	60-70	Adenocarcinoma	T3N0M0	III	Malignant	4	++
16	70-80	Adenocarcinoma	-	-	Malignant	-	++
17	>80	Adenocarcinoma	-	-	Malignant	3	++
18	70-80	Adenocarcinoma	T3N0M0	III	Malignant	4	+++

19	60-70	Adenocarcinoma(sparse)	T3N1M0	IV	Malignant	-	+
20	60-70	Adenocarcinoma	T2N0M0	IIA	Malignant	4	++
21	60-70	Adenocarcinoma	T3N0M1	IV	Malignant	4	+
22	60-70	Adenocarcinoma	T2N0M0	IB	Malignant	4	++
23	70-80	Adenocarcinoma	T2N0M0	IIA	Malignant	4	-
24	70-80	Adenocarcinoma	-	-	Malignant	4	+
25	70-80	Adenocarcinoma	T2N0M0	II	Malignant	5	+
26	50-60	Adenocarcinoma	T2N0M0	II	Malignant	5	++
27	50-60	Adenocarcinoma	-	-	Malignant	5	-
28	60-70	Adenocarcinoma	-	-	Malignant	3	-
29	70-80	Adenocarcinoma	T2N0M0	II	Malignant	4	++
30	60-70	Adenocarcinoma	T2N0M0	II	Malignant	4	++
31	60-70	Adenocarcinoma	T2N0M0	II	Malignant	4	+++
32	70-80	Adenocarcinoma	T2N0M0	I	Malignant	4	+
33	70-80	Adenocarcinoma	T4N1M1	IV	Malignant	4	+
34	60-70	Adenocarcinoma	T2N0M0	II	Malignant	5	-
35	60-70	Adenocarcinoma	-	-	Malignant	4	++
36	70-80	Adenocarcinoma	T3N0M0	III	Malignant	4	+/-
37	>80	Adenocarcinoma	T3N0M0	III	Malignant	5	+
38	70-80	Adenocarcinoma	T3N0M0	III	Malignant	4	-/+
39	60-70	Adenocarcinoma	T2N0M0	II	Malignant	4	-
40	>80	Adenocarcinoma	T3N1M0	IV	Malignant	4	+
41	70-80	Adenocarcinoma	T1N0M0	I	Malignant	4	++
42	60-70	Adenocarcinoma	T2N0M0	II	Malignant	5	+
43	70-80	Adenocarcinoma	T2N0M0	II	Malignant	5	+

44	<30	Adenocarcinoma	T3N0M0	III	Malignant	4	+
45	60-70	Adenocarcinoma	T3N0M0	III	Malignant	3/-	++/*
46	60-70	Adenocarcinoma	T3AN0M0	III	Malignant	-/3	-
47	70-80	Adenocarcinoma	T2bN0M0	IIA	Malignant	3	+
48	60-70	Adenocarcinoma	T2AN0M0	II	Malignant	5	-/+
49	60-70	Adenocarcinoma	T3AN0M0	III	Malignant	5	+
50	60-70	Adenocarcinoma	T3N1M0	IV	Malignant	5	+
51	70-80	Adenocarcinoma	T3N0M0	III	Malignant	4	++
52	50-60	Adenocarcinoma	-	-	Malignant	4	+
53	70-80	Adenocarcinoma	T3N0M0	III	Malignant	5	-/+
54	70-80	Adenocarcinoma	T4N1M1	IV	Malignant	5	++
55	70-80	Adenocarcinoma	T2N1M1C	IV	Malignant	5	+
56	70-80	Adenocarcinoma	-	-	Malignant	-	-
57	60-70	Adenocarcinoma	T3N0M0	III	Malignant	3	++
58	60-70	Adenocarcinoma	T2N0M0	II	Malignant	4	+
59	<30	Adenocarcinoma	T2N0M0	II	Malignant	3	++
60	50-60	Adenocarcinoma	T3N0M0	III	Malignant	5	-
61	70-80	Adenocarcinoma	-	-	Malignant	5	-
62	70-80	Adenocarcinoma	-	-	Malignant	-	*
-63	70-80	Adenocarcinoma	T3N1M0	IV	Malignant	5/-	-
64	70-80	Adenocarcinoma	-	-	Malignant	5	+
65	70-80	Adenocarcinoma	T2N0M0	II	Malignant	5	+/-
66	60-70	Adenocarcinoma	-	-	Malignant	5	-/+
67	70-80	Adenocarcinoma	T2BN0M0	IIA	Malignant	5	-
68	60-70	Adenocarcinoma	-	-	Malignant	-/5	-/*

69	60-70	Adenocarcinoma	T2N0M0	IIA	Malignant	-	*/+
70	70-80	Adenocarcinoma	T3N1M1C	IV	Malignant	5	+/-
71	60-70	Adenocarcinoma	T2N0M0	II	Malignant	-/5	+/-
72	60-70	Adenocarcinoma	T2N0M0	II	Malignant	5	-
73	60-70	Adenocarcinoma	-	-	Malignant	5	-/+
74	60-70	Adenocarcinoma	T2N0M0	II	Malignant	5	+/-
75	60-70	Adenocarcinoma	T2N0M0	II	Malignant	5	-
76	80>	Adenocarcinoma	T2N0M0	II	Malignant	5	-/+
77	70-80	Adenocarcinoma	T3N0M0	III	Malignant	5	-
78	60-70	Adenocarcinoma	-	-	Malignant	5	-
79	70-80	Adenocarcinoma	T4N0M0	IV	Malignant	5	-
80	80>	Adenocarcinoma	T3N0M0	III	Malignant	5	-
81	<30	Adjacent normal prostate tissue	-	-	NAT	-	+
82	30-50	Adjacent normal prostate tissue	-	-	NAT	-	-
83	<30	Adjacent normal prostate tissue	-	-	NAT	-	+
84	50-60	Adjacent normal prostate tissue	-	-	NAT	-	+
85	60-70	Adjacent normal prostate tissue	-	-	NAT	-	-
86	60-70	Adjacent normal prostate tissue	-	-	NAT	-	-/+
87	50-60	Adjacent normal prostate tissue	-	-	NAT	-	++
88	60-70	Adjacent normal prostate tissue	-	-	NAT	-	+/++
89	30-50	Prostate tissue	-	-	Normal	-	+
90	30-50	Prostate tissue	-	-	Normal	-	++
91	30-50	Prostate tissue	-	-	Normal	-	*/+
92	30-50	Prostate tissue	-	-	Normal	-	+
93	<30	Prostate tissue	-	-	Normal	-	+

94	30-50	Prostate tissue	-	-	Normal	-	+
95	30-50	Prostate tissue	-	-	Normal	-	++
96	<30	Prostate tissue	-	-	Normal	-	++
